# Oxidative Stress and Aging Diseases

**DOI:** 10.1155/2014/569146

**Published:** 2014-05-12

**Authors:** Peter X. Shaw, Geoff Werstuck, Yan Chen

**Affiliations:** ^1^Department of Ophthalmology, University of California San Diego, La Jolla, CA 92093, USA; ^2^Thrombosis and Atherosclerosis Research Institute (TAARI), David Braley Research Institute, Hamilton General Hospital Campus, McMaster University, Hamilton, ON, Canada; ^3^Institute for Nutritional Sciences, Shanghai Institutes for Biological Sciences (SIBS), Chinese Academy of Sciences, Shanghai 200031, China

Many aging disorders, including atherosclerosis, Alzheimer's disease, diabetes, and age-related macular degeneration (AMD), result from years of combinatory impact of environmental assaults and genetic susceptibility. As a major environmental risk factor, oxidative stress, which can be enhanced by smoking and dietary habits, is known to increase the risk for many diseases associated with aging. Systemic oxidative stress not only results in accumulation of reactive oxygen species (ROS), but also damages DNA or modifies DNA structures at an epigenetic level, altering expression of genes that associate with the aging process. Local oxidative stress and ROS can affect cell signaling as well as trigger apoptosis, cellular senescence, inflammation, and even necrosis. Elucidation of mechanisms and pathways, through which oxidative stress modulates these cellular and molecular processes and affects age-related disease pathologies, is the major focus of this special issue. To respond to our call, authors presented their up-to-date studies and results in basic and translational studies of oxidative stress on diseases of aging.

ROS and derivatives of lipid peroxidation have been used as biomarkers to diagnose many aging diseases and monitor their progressions. In this issue, O. O. Erejuwa et al. reviewed the physiological and pathophysiological roles of ROS in cancer patients and highlighted evidence demonstrating other potential applications of lipid peroxidation products in cancer treatment, including the use of lipid peroxidation products as a biomarker for a diagnostic tool to predict the chances of cancer recurrence and to monitor treatment progress or how well cancer patients respond to therapy. A. Anand et al. estimated the levels of superoxide dismutase1 (SOD1) in patients with age-related macular degeneration (AMD) and examined the roles of oxidative stress, smoking, hypertension, and other factors involved in the pathogenesis of AMD. X. Xiao et al. observed the relationship between changes in renin-angiotensin-aldosterone system (RAAS) activity and blood plasma glucose after an administration of hydrochlorothiazide (HCTZ) for one year in patients with hypertension. Changes in RAAS activity were correlated with changes in plasma glucose levels after one year of HCTZ therapy.

Cataract is a common age-related eye disorder. D. Chang et al. investigated the activity of antioxidative enzymes and the products of oxidative stress in patients with age-related cataracts and compared the findings with those in healthy control subjects. They found that oxidative stress is an important risk factor in the development of age-related cataract, and augmentation of the antioxidant defence systems may be beneficial in preventing or delaying cataractogenesis.

To respond to our call for advances in genetics/epigenetics of aging related diseases, A. A. Rahman et al. determined the differential gene expression profile in genetically related individuals but at different age groups using genome-wide microarray analysis. Their results suggested that systemic telomere maintenance, metabolism, cell signalling, and redox regulation may be important for individuals to maintain their healthy state with age progression and that these processes play an important role in the determination of a healthy life span.

In other exciting papers, E. Palli et al. assessed the incidence of contrast-induced nephropathy (CIN) in critically ill patients with stable renal function who underwent computed tomography with intravenous contrast media and found that older critically ill patients are more prone to develop renal dysfunction after the intravenous infusion of contrast agents in relation to their younger counterparts. M. Arciello et al. investigated the roles of mitochondria and metals in hepatitis C virus- (HCV-)related oxidative stress, highlighting the need to reconsider their deregulation in the HCV-related liver damage and in the antiviral management of patients.

In summary, this special issue covers a wide range of topics addressing the problems linking oxidative stress to age-related disorders and their potential treatments. These researchers not only enrich our understanding of how oxidative stress plays an important role in the initiation and progression of these diseases, but also provide evidence on antioxidant therapies for aging diseases in both experimental and clinical settings.

